# Environmental Enteric Dysfunction Is Associated With Poor Linear Growth and Can Be Identified by Host Fecal mRNAs

**DOI:** 10.1097/MPG.0000000000001315

**Published:** 2016-10-24

**Authors:** Maria Isabel Ordiz, Nurmohammad Shaikh, Indi Trehan, Ken Maleta, Jennifer Stauber, Robert Shulman, Sridevi Devaraj, Phillip I. Tarr, Mark J. Manary

**Affiliations:** ∗ Department of Pediatrics, Washington University, St Louis, MO; † College of Medicine, University of Malawi, Blantyre, Malawi; ‡ Department of Pathology, Washington University, St Louis, MO; § Department of Pediatrics, Baylor College of Medicine, Houston, TX.

**Keywords:** droplet digital PCR, dual sugar absorption test, environmental enteric dysfunction, host fecal transcripts, random forest modeling, stunting

## Abstract

**Objective:**

Environmental enteric dysfunction (EED) can be assessed by the lactulose:mannitol (L:M) test. Our objective was to determine if selected host fecal transcripts were correlated with EED, and whether transcripts and clinical characteristics could be used to predict EED in rural African children.

**Methods:**

Demographic and sanitation characteristics, along with L:M testing and host fecal transcript analyses from 798 asymptomatic Malawian children aged 12 to 61 months were compared with linear growth over the subsequent 3 months. Fecal host mRNA analysis included quantification of expression of 18 transcripts associated with L:M. Permeability was categorized as normal (L:M ≤ 0.15), moderate (0.15<L:M<0.45) and severe (L:M ≥ 0.45), and random forest predictive models were created.

**Results:**

L:M was inversely correlated with linear growth over the subsequent 3 months (*r* = −0.32, *P* < 0.001) and severe EED was associated with stunting (*P* < 0.0001). Age younger than 24 months, weight-for-height *z* score <0, domesticated animals in the child's sleep environment, lack of a pit latrine combined with a potentially contaminated water source, and a recent history of diarrhea were associated with severe EED. A random forest model using CD53, HLA-DRA, MUC12, and TNF was 84% sensitive for severe EED and 83% sensitive for no EED.

**Conclusions:**

Selected host fecal transcripts can be used in a random forest model as a noninvasive biomarker for categories of EED in rural African children.

## Abstract 2

Supplemental Digital Content is available in the text

**What Is Known**

- Environmental enteric dysfunction is associated with reduced linear growth/stunting.
- The cumbersome lactulose:mannitol test is used to assess environmental enteric dysfunction.
- A noninvasive biomarker to identify environmental enteric dysfunction is needed.

**What Is New**

- Risk factors for environmental enteric dysfunction in rural African children are identified.
- Quantification of fecal host mRNAs in a random forest model shows promise as a noninvasive method to identify children with environmental enteric dysfunction.

Optimal gut health includes as the ability of the intestines to absorb all necessary dietary nutrients while mounting responses to limit dissemination of inflammatory microbes and their products from the lumen, so as to avert local and systemic inflammation. Environmental enteric dysfunction (EED) is the most pervasive condition associated with poor gut health worldwide, which is prevalent in rural African children and is associated with stunting (1–3).

Tissue-dependent assessment of gut health involves visualization of the mucosa and biopsy (4). Endoscopy is, however, an invasive and expensive procedure that is poorly suited to mass screening, or to frequent reassessment in individuals. The dual sugar absorption test is a widely used alternative, in which lactulose and mannitol are ingested, and urines are collected during the subsequent several hours. Disrupted cell junctions allow lactulose, a disaccharide, and mannitol, a monosaccharide, to be passively absorbed, providing a measure of gut epithelial integrity. Mannitol is also absorbed across cell membranes, which is measure of the surface area of the intestinal tract. Once absorbed, these sugars are excreted intact in the urine. The ratio of urinary lactulose to mannitol (L:M) indicates the degree of epithelial disruption in the small bowel, and, by extension, poor gut health (5). The L:M test is theoretically sound and often used, but cumbersome to administer.

Stool contains exfoliated enterocytes, representing gut mucosal tissue. Stool specimens are acquired noninvasively. Fecal extractions have been used to analyze expression of individual host transcripts by droplet digital PCR (ddPCR) (6,7). Measurement of host fecal transcripts is challenging because human mRNA is estimated to be <1% of total fecal RNA, which is predominantly of microbial and ribosomal origin. mRNA in feces is also relatively degraded, and quantification can be further hampered by coextracted inhibitors, but methods to overcome these limitations have recently been developed (7).

Here we use the L:M test as the standard to assess gut health in rural Malawian children, determine the relation between L:M and linear growth, and compare a panel of host fecal transcripts and clinical characteristics to this standard.

## METHODS

### Subjects

Among rural Malawian children 12 to 61 months of age who participated in 1 of 3 clinical studies, 798 did not receive an intervention and were therefore eligible for inclusion in this biomarker study (8–10). Children with severe acute malnutrition, diarrhea within the last 3 days, congenital abnormalities, and chronic debilitating illnesses were excluded. Enrolled children were from families of rural subsistence farmers, consumed water from wells or boreholes, lived in unelectrified mud huts, and were at high risk for EED. Ethical approval was obtained from the University of Malawi, Baylor College of Medicine, and Washington University in St Louis.

### Study Design

This was an observational study to determine whether a single L:M predicts subsequent linear growth in these rural African children; which dietary, demographic, and household sanitation practices are associated with L:M; and the extent to which fecal host mRNAs predict L:M. The participants had originally been enrolled in 1 of 3 clinical studies, which included L:M testing using a uniform, controlled, and standardized method. One of the studies monitored the growth of twins in 4 villages monthly, with L:M testing conducted at a single point in time (8). The other 2 studies were randomized, double-blind, placebo-controlled clinical trials to ameliorate EED and from these studies only the data collected at the time of enrollment from children assigned to placebo group were included in these analyses (9,10). Thus the data do not represent a longitudinal cohort, and L:M testing was not available from >1 time for any given child.

Using data from these 3 studies, the primary outcomes evaluated were the ability of L:M to predict subsequent linear growth in a linear regression model, identification of clinical and environmental risk factors for EED as defined by an abnormal L:M, and the sensitivity and specificity of random forest modeling of sets of fecal host transcripts to predict the severity of EED.

### Participation

Information regarding the demographics, dietary intake, and household sanitation practices information was collected using a standard questionnaire from the child's primary caretaker. Length and weight were measured as previously described (9,10).

All of the subjects underwent a carefully conducted L:M test with adequate urine collection and sugar excretion (11,12). Caregivers were directed not to feed the child for 12 hours before participation. Children were given lactulose (5 g) and mannitol (1 g) dissolved in 20 mL of water. All urine was collected from the time of sugar ingestion through at least 4 hours after sugar ingestion in containers with 10-mg merthiolate, to limit bacterial degradation of the sugars. Children were encouraged to drink water to facilitate urination and mothers were instructed not to breast-feed their children during this time. The total urine volume was measured and a 4-mL aliquot was transferred into cryovials, flash frozen on site, and transported frozen (−80°C) to Baylor College of Medicine.

Fresh stool specimens were collected on site before completion of the L:M testing using a small, clean nonabsorbent, plastic diaper. The stools were immediately transferred to cryovials and flash frozen in liquid nitrogen, without buffers, enzymes, or preservative solutions. Samples were transferred to a −80°C freezer and transported to Washington University (St Louis, MO) at −80°C, where they were then processed and analyzed for human fecal mRNA.

### Laboratory Analyses

Concentrations of lactulose and mannitol in the urine specimens were analyzed by high-performance liquid chromatography (HPLC) using a modification of the method of Catassi et al (13,14). The assays are sensitive to 1 μg/mL lactulose and mannitol and the coefficient of variation is ≤5% (15). Because of concerns raised in the literature of the accuracy of HPLC measurements of lactulose and mannitol (16), 115 samples were sent to the laboratory of Dr William Faubion at Mayo Clinic (Rochester, MN) and tested using liquid chromatography-tandem mass spectrometry (Applied Biosystems-MDS SCIEX, Foster City, CA) (16). Urines were mixed with 250 μL of internal standard, ^13^C labeled mannitol and lactulose, and chromatographic separation is achieved using a CARBOSep COREGEL 87C column from Transgenomic (Transgenomic, Omaha, NE) at 0.6 mL/min and 83^o^C. Intra-assay coefficients of variation for mannitol ranged from 2.6% to 4.1% for levels at 1.52, 35.8, 146, and 458 μg/mL and lactulose coefficients of variation ranged from 3.0% to 6.4% for levels at 0.6, 4.65, 38.9, and 143 μg/mL.

Host fecal mRNAs were analyzed using a conservative method for host mRNA isolation and ddPCR as previously described (6,7). This method uses glyceraldehyde 3-phosphate dehydrogenase as an internal standard to normalize all transcript measurements, which reduces variations in measurement values when samples are run in different settings or in different conditions. Eighteen transcripts correlated with L:M (7) were eligible for incorporation into the random forest model.

### Data Analysis

Clinical and laboratory data were aggregated from the enrollment data from the 3 clinical studies. Height-for-age *z* score (HAZ) and weight-for-height *z* score (WHZ) were determined using the 2006 World Health Organization Multicenter Growth Reference Study child growth standards (17). L:M was calculated as the ratio of the lactulose to mannitol concentrations in the urine.

To identify children at greatest risk for stunting and create a categorical random forest model to predict EED, 3 categories of L:M were designated; no EED as L:M ≤ 0.15, moderate EED as 0.15 < L:M < 0.45 and severe EED as L:M ≥ 0.45. The L:M value chosen to represent no EED is based on measurements made in healthy individuals in Europe and North America (18,19). Severe EED was designated to be L:M > 0.45 because this corresponds to L:M measurements in children with Crohn disease in remission and in celiac disease (20,21). No EED constituted 18% of the study population, moderate EED 66% of the study population, and severe EED 17% of the study population. The demographic, dietary, anthropometric, and sanitation practice characteristics were compared for these 3 categories of EED using a one-way ANOVA (SPSS22, Chicago, IL).

Linear growth measurements over the 3 months subsequent to specimen acquisition were available from children in 2 of the 3 studies (8,10); change in HAZ (ΔHAZ) was calculated for each child from those 2 studies and these data were used to create a stepwise, backward linear regression model to predict ΔHAZ. L:M was the primary independent variable; other covariates were the child's age, sex, WHZ, HAZ, whether his/her mother was the primary caregiver, whether the father was alive, number of siblings, number of individuals that sleep in the same room as the child, roofing material, bicycle ownership, whether animals sleep in the house with the child, whether water is from a clean source, whether the family uses a pit latrine, household food insecurity score (22), dietary diversity score (23), number of times per day animal source foods are consumed, and diarrhea reported 4 to 7 days before L:M testing. Covariates were considered significant if *P* < 0.05. The third study in our dataset included children with L:M, collection of clinical and dietary information and fecal specimens; however, the duration of follow-up was only 7 weeks, so reliable linear growth data were not available (9).

### Modeling

We chose random forest modeling as the machine-based learning method of modeling because it is the most appropriate and powerful for determining categorical outcomes, in our case no EED, moderate EED, and severe EED. Random forest modeling creates a set of computer-generated decision trees from a set independent variables, in our case the copy numbers of a set host transcripts, to designate the child into an EED category (10). Each tree is given a “vote” and the category with the most votes for a given child's matrix is the assigned EED category. Random forest modeling was performed using the rf package in R (*www.r-project.org*, Vienna, Austria) and default settings. Cross validation of models was done by removing 30 children from the testing and training sets, and testing the accuracy of the model in this naïve group. This method of random forest modeling was chosen because the desired output of the model was the categorical severity of EED, and fine-grained numerical L:M prediction was not felt to be clinically meaningful.

## RESULTS

### Study Subjects and L:M Testing

Of the 798 children included in the study, 140 (18%), 524 (66%), and 134 (17%) had no, moderate, or severe EED, respectively (Table 1).

**TABLE 1:** Characteristics of the rural Malawian children categorized by EED severity^†^

Characteristic	No EED L:M ≤ 0.15 (n = 140)	Moderate EED 0.15 < L:M< 0.45 (n = 524)	Severe EED L:M ≥ 0.45 (n = 134)	*P*^‡^
Age, mo	37.7 ± 10.5	31.4 ± 11.3	25.4 ± 10.2	<0.001^*^
Female, n (%)	65 (46%)	262 (50%)	57 (43%)	0.28
Mid-upper arm circumference, cm	15.2 ± 1.2	14.9 ± 1.2	14.4 ± 1.3	<0.001^*^
Weight-for-height *z* score	0.28 ± 0.95	0.06 ± 0.91	−0.38 ± 1.05	<0.001^*^
Height-for-age *z* score	−2.5 ± 1.2	−2.3 ± 1.2	−2.1 ± 1.2	0.05
Stunted,^§^ n (%)	92 (66%)	307 (59%)	70 (52%)	0.08
Caregiver is mother, n (%)	131 (94%)	498 (95%)	127 (95%)	0.73
Father is alive, n (%)	132 (94%)	512 (98%)	132 (99%)	0.06
Siblings	3.7 ± 1.7	3.5 ± 1.8	2.8 ± 1.9	<0.001^*^
Individuals that sleep in same room as child	3.1 ± 1.6	3.3 ± 1.4	3.2 ± 1.0	0.28
Home with a metal roof, n (%)	37 (26%)	114 (22%)	35 (26%)	0.36
Family owns bicycle, n (%)	83 (59%)	305 (58%)	68 (51%)	0.25
Animals sleep in house, n (%)	40 (29%)	219 (42%)	56 (42%)	0.014^*^
Water from a clean source, n (%)	72 (51%)	376 (72%)	91 (68%)	<0.001^*^
Uses a pit latrine, n (%)	70 (50%)	314 (60%)	56 (42%)	<0.001^*^
Child does not use pit latrine or clean water, n (%)	31 (22%)	178 (34%)	67 (50%)	<0.001^*^
Household Food Insecurity Access Score^||^	2.8 ± 3.9	3.2 ± 3.8	4.0 ± 3.7	0.03^*^
Dietary diversity score^¶^	4.6 ± 1.1	4.3 ± 1.2	4.0 ± 1.2	<0.001^*^
Consumes animal source foods, times per day^#^	1.6 ± 1.4	1.6 ± 1.3	2.0 ± 1.8	0.012^*^
Diarrhea in last days, n (%)	11 (8%)	101 (19%)	43 (32%)	<0.001^*^
Lactulose:mannitol	0.11 ± 0.04	0.27 ± 0.08	0.69 ± 0.25	<0.001^*^
Lactulose, % excreted	0.2 ± 0.1	0.4 ± 0.2	0.7 ± 0.4	<0.001^*^
Mannitol, % excreted	8.3 ± 4.9	6.8 ± 3.3	5.5 ± 2.6	<0.001^*^

EED = environmental enteric dysfunction; SD = standard deviation.

^*^P < 0.05.

^†^Data are expressed as means ± SD for continuous measures or counts (percentages) for categorical measures.

^‡^For continuous characteristics *P* value calculated with one-way ANOVA with Tukey's correction and for categorical characteristics *P* value calculated using Chi-square test in a 2 × 3 table. Main effects and interactions were considered significant at *P* < 0.05.

^§^Defined as height-for-age *z* score <−2.

^||^Household Food Insecurity Access Score measures the degree of food insecurity in the household in the past 4 weeks for the 9 food insecurity related conditions. Range 0 to 27 with higher scores representing more food insecurity (22).

^¶^Range 0 to 7, higher score more dietary diversity (23).

^#^Animal source foods defined as foods containing meat, milk, fish, poultry, or eggs.

The HPLC analytical method used to determine lactulose and mannitol concentrations was validated in 115 urine samples using liquid chromatography-tandem mass spectrometry, 6 of 115 (5%) of the L:M values were discordant, defined as >25% difference in measurements using the 2 methods. Using all data points, the *r* values between the 2 methods were 0.88 for L:M and 0.92 for lactulose (*P* < 0.001). A Bland-Altman plot shows average differences between the 2 methods of 0.1% for L and 1% for M, which is excellent agreement (Supplemental Digital Content, Fig. 1).

### Clinical Associations With Categories of Environmental Enteric Dysfunction

Age younger than 24 months, WHZ < 0, animals sleeping in the same room as the child, use of a potentially contaminated water source combined with the absence of a pit latrine in the household, and diarrhea 4 to 7 days before L:M testing are the characteristics associated with EED (Table 1). When 4 or more of these characteristics were present, only 3% of such children had no EED, whereas 46% had severe EED. These values contrast with the 18% and 17% background rates of no or severe EED observed in the study population, respectively (*P* = 0.0003). We could, however, find no combination of clinical characteristics that predict severe EED from either moderate EED or no EED with >65% sensitivity.

### L:M and Linear Growth

**FIGURE 1:**
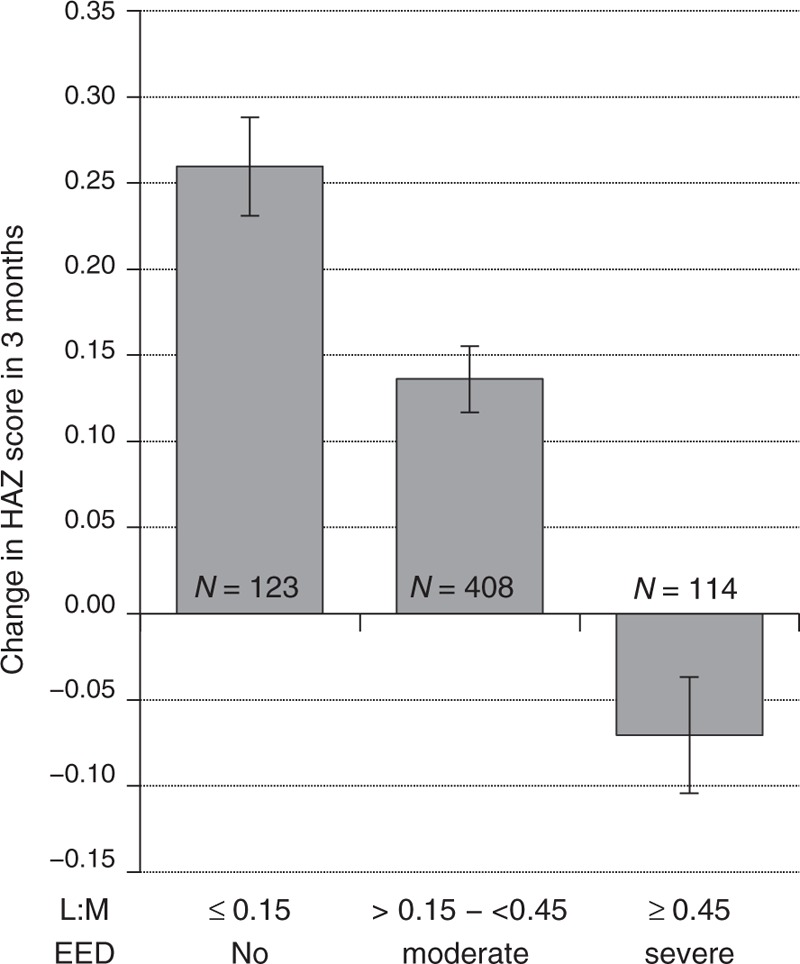
Change in height-for-age *z* scores in the 3 months after L:M testing. L:M values are categorized as no EED, moderate EED, or severe EED. Data expressed as mean ± SEM. Differences between any of the 3 categories were statistically significant, (*P* < 0.0001), using one-way ANOVA. ANOVA = analysis of variance; EED = environmental enteric dysfunction; HAZ = height-for-age *z* score; L:M = lactulose to mannitol; SEM = standard error mean.

L:M was correlated with ΔHAZ (Pearson correlation coefficient = −0.27 (*P* < 0.001) and Spearman correlation coefficient = −0.32 (*P* < 0.001)). Severe EED was associated with decreased ΔHAZ in the subsequent 3 months (Fig. 1). Linear regression modeling identified L:M as a significant predictor of ΔHAZ (Table 2).

**TABLE 2:** Characteristics that predict change in height-for-age over subsequent 3 months^∗^

Model	Standardized coefficients beta	*t*	*P*
Constant		−3.226	0.001
Lactulose:mannitol	−0.140	−3.971	<0.001
Age, mo	0.147	3.913	<0.001
Height-for-age, *z* score	−0.352	−10.429	<0.001
Weight-for-height, *z* score	0.197	5.705	<0.001
Household Food Insecurity Access Scale^†^	−0.106	−3.009	0.003

^*^Determined by developing a stepwise, backward linear regression model (*r* = 0.564, *P* < 0.001), only covariates with *P* < 0.05 listed in table. All characteristics included in Table 1 were used in the prediction model.

^†^Household Food Insecurity Access Score measures the degree of food insecurity in the household in the past 4 weeks for the 9 food insecurity related conditions. Range 0 to 27 with higher scores representing more food insecurity (22).

### Random Forest Models to Associate Host Fecal mRNA With L:M

Eighteen transcripts of interest recently identified as being associated with L:M were evaluated for their association with EED (Table 3) (7). Random forest modeling identified 7 of these transcripts as important in models to predict EED.

**TABLE 3:** Summary of 18 transcripts correlated with L:M, shaded are the 7 transcripts identified as significant predictors of L:M in random forest modeling

Gene symbol	Gene description	Primary function	Mean ± SD (n)	Transcript concentration^*^; median (25th, 75th percentiles)	Spearman's *r* with L:M (*P* value of *r*)
ACP1	Acts on tyrosine phosphorylated proteins, low-MW aryl and acyl phosphates. Isoform 3 does not possess phosphatase activity		0.009 ± 0.009 (378)	0.007 (0.005, 0.011)	−0.104 (0.043)
AQP9	Forms a transmembrane channel. Mediates passage of noncharged solutes including carbamides, polyols, purines, and pyrimidines	Transporter activity	0.145 ± 0.318 (73)	0.065 (0.019, 0.150)	0.299 (0.01)
BIRC3	Regulates caspases and apoptosis, modulates inflammatory signaling and immunity, mitogenic kinase signaling, and cell proliferation	Inflammatory response	0.169 ± 0.177 (542)	0.120 (0.077, 0.196)	−0.125 (0.004)
CD53	Mediates signal transduction promoting cell development. Complexes with integrins. Mutations in this gene result in immunodeficiency	Cell adhesion	0.076 ± 0.124 (324)	0.035 (0.013, 0.091)	0.168 (0.002)
CDX1	Caudal type homeobox 1. Plays a role in the terminal differentiation of the intestine	Intestinal differentiation	0.047 ± 0.346 (562)	0.027 (0.016, 0.042)	−0.166 (<0.001)
DECR1	Enzyme of β-oxidation. It participates in the metabolism of unsaturated fatty enoyl-CoA esters	Fatty acid metabolism	0.041 ± 0.027 (85)	0.035 (0.022, 0.047)	0.220 (0.043)
DEFA6	Has antimicrobial activity against Gram-negative and Gram-positive bacteria. Protects cells against infection with HIV-1	Viral response	0.090 ± 0.142 (443)	0.041 (0.016, 0.099)	0.118 (0.013)
HLADRA	Binds peptides from antigens that access the endocytic route of antigen presenting cells and presents them on the cell surface for T cells	Adaptive immune response	0.220 ± 0.170 (567)	0.174 (0.103, 0.281)	−0.125 (0.003)
IFI30	Lysosomal thiol reductase reduces disulfide bonds, unfolds proteins destined for lysosomal degradation. Active in antigen processing	Antigen processing	0.309 ± 0.611 (73)	0.168 (0.081, 0.288)	0.264 (0.024)
LYZ	Lysozymes have primarily a bacteriolytic function; those in tissues and body fluids are associated with the monocyte-macrophage system	Bacterial response	0.080 ± 0.112 (324)	0.047 (0.022, 0.095)	0.218 (<0.001)
MUC12	Mucin 12. A protein in gastrointestinal mucous layer, involved in epithelial cell protection, cell adhesion, and epithelial cell growth. Stimulated by cytokines	Epithelial barrier function	0.447 ± 0.499 (319)	0.294 (0.163, 0.539)	−0.228 (<0.001)
PIK3AP1	Signaling adapter in B-cell development. Links Toll-like receptor signaling to PI3K activation, reduces inflammatory cytokines	Inflammatory response	0.199 ± 0.420 (77)	0.048 (0.022, 0.121)	0.249 (0.029)
REG1A	Acts as an inhibitor of spontaneous calcium carbonate precipitation. Associated with intestinal, brain, and pancreas regeneration	Regeneration of epithelial cells	0.114 ± 0.268 (622)	0.042 (0.018, 0.107)	0.173 (<0.001)
REG3A	Bactericidal lectin that acts against Gram-positive bacteria and mediates bacterial killing by binding to surface-exposed carbohydrate moieties	Bacterial response	0.123 ± 0.233 (344)	0.040 (0.015, 0.100)	0.183 (0.001)
S100A8	Calprotectin, a cation-binding protein that regulates inflammation and immune response. Induces neutrophil chemotaxis and adhesion	Innate immune response	0.979 ± 1.738 (550)	0.386 (0.154, 1.169)	0.096 (0.025)
SELL	Cell surface adhesion protein. Promotes initial tethering and rolling of leukocytes in endothelia	Cell adhesion	0.052 ± 0.134 (80)	0.009 (0.003, 0.038)	0.295 (0.008)
SI	Sucrase isomaltase. A disaccharidase that plays an important role in carbohydrate digestion	Carbohydrate digestion	0.114 ± 0.895 (753)	0.017 (0.008, 0.036)	−0.104 (0.004)
TNF	Cytokine that binds to TNFRSF1A/TNFR1. Secreted by macrophages, potent pyrogen, promotes cell death. Induces IL-12 in dendritic cells	Innate immune response	0.008 ± 0.015 (751)	0.004 (0.002, 0.008)	−0.149 (<0.001)

SD = standard deviation.

^*^Expressed as copies/copy GAPDH.

A random forest model to identify children with severe EED was created using CDX1, HLA-DRA, MUC12, REG1A, S100A8, and TNF, and the model was 84% sensitive and 73% specific (n = 284, node size = 4, max node = 20, mtry = 3). Validation of model with 30 samples removed from the model creation exercises yielded 80% sensitivity and 72% specificity. A random forest model to discriminate children without EED from those with severe EED was created using TNF, HLA-DRA, MUC12, and CD53 and found to be 84% sensitive for severe EED and 83% sensitive for no EED (n = 284, node size = 4, max node = 20, mtry = 3). Validation of this model with 30 samples removed from the model creation exercises resulted in a prediction with 83% sensitivity for the identification of children with severe EED and 86% sensitivity for identification of children without EED.

## DISCUSSION

In rural Malawians aged 12 to 61 months, increased gut permeability, as measured by L:M, is a predictor of linear growth faltering. Severe EED can be predicted by a small number of host fecal mRNAs using random forest modeling with 80% to 85% sensitivity.

The primary limitation of the present study is that the use of the L:M test was not extended to younger children. Growth faltering in the first year of life is of great interest in the global health community, and there is often the assumption that EED plays a causal role infant stunting. Identification of a biomarker for EED in infants would be a powerful tool in elucidating the role of EED in stunting and identifying children who may benefit from an intervention to ameliorate stunting. L:M testing in such infants could be, however, problematic because lactulose can cause osmotic diarrhea and infants may be more compromised by the consequent to fluid loss. In addition, complete urine collections are more difficult in infants than toddlers. Another limitation is that the association of L:M and growth faltering is based on a single L:M measurement and a single linear growth measurement 3 months later, a more powerful methodology to implicate abnormal L:M in growth faltering would be to test a child every 3 months over 1 to 2 years and associate these multiple L:M measurements with change in length.

We are encouraged by the demonstration that host fecal transcripts can be used in a machine learning model to predict EED with >80% sensitivity. Stool collections are noninvasive. Our group has done extensive work investigating the transcriptome in EED, and we recognize that none transcript will serve as biomarker with sufficient sensitivity and specificity (24). This technology, built on iterative design using expert opinion and nonbiased high-density microarray nomination of candidate transcripts, however, enables adaptive mRNA biomarkers to be constructed on a cohort and geographic-specific basis. Moreover, these disease and population-tailored readouts can be determined on materials that are handled with minimal processing on site and uniform purification technology downstream.

A caveat regarding the diagnostic characterization of EED as used in the present study is that we focused on small bowel dysfunction as the canonical lesion in EED. The more general constellation of growth faltering and increased systemic inflammation in an asymptomatic child living in an unsanitary environment, which is sometimes described as environmental enteropathy (without the dysfunction component), certainly includes children with a spectrum range of L:M measurements. In such children the host fecal mRNAs identified in the present study may be less likely to constitute an adequate biomarker of the general environmental enteropathy syndrome. Their utility in identifying those with enteropathy who may progress to stunting, however, seems worthy of further pursuit.

The primary biomarker for EED is at present the L:M test. No other biomarker has been used as an outcome in a clinical trial to ameliorate EED or is associated with change in length (18,25–28). The L:M test stresses upper intestinal integrity; a large, inert osmotic load is added to the lumen and evidence of excessive paracellular leakage is sought. This theoretically sound test is compromised by physiologic and technical issues. The dual sugar load is likely to create fluid shifts between intestine tissue and gut lumen (reverse solute drag) (29), evoke an immunologic stress response (24), and temporarily alter gut microbial communities (30). For these reasons, repeated L:M tests performed on consecutive days may well yield different results. Moreover, the cumbersome nature of a several hour urine collection from a young child, which is required for a successful L:M test, is obviated with the use of host fecal transcripts. In addition, there is endogenous mannitol in human urine (31). Measurement of host fecal transcripts does not perturb gut biology, and for this reason, could be used to measure EED repeatedly and frequently to assess intestinal health.

In the present study, the cost of conducting an L:M test was about $90, whereas the host transcript test with 4 transcripts costs $35.

We acknowledge that a normal L:M value in a variety of populations are not available, nor is an estimate of the effect size of a change in L:M on linear growth (4). In part this is because the L:M test is not conducted in a uniform manner, different doses of sugars are used, timing of urine collections vary, and assay technologies differ. These issues necessitate that our categories of no, moderate and severe EED be defined empirically. These categories are, however, similar to other studies that have used the L:M test (4), and are useful in understanding the causes of stunting in rural African children.

An alternative noninvasive approach for EED is to measure fecal host proteins (32). Although this has been described in the literature, it has not been used as an outcome measure in any clinical studies. Detection of a protein in feces is contingent upon significant quantities of the protein being secreted into the extracellular space and the secreted protein remaining largely intact as it passes along the intestinal tract, which is unusual given the abundance of proteases present in the intestinal lumen from the host and its microbiota. α1-Antitrypsin, calprotectin, myeloperoxidase, neopterin, and lithostathine 1β are proteins that are suitable for measurement in fecal samples and have been described as EED biomarkers (32–37). Although commercial enzyme immunoassays are available for each of these proteins, their use is limited by variable reliance on polyclonal antibodies (which reduce specificity and may contribute to batch to batch variability), cost, the mass of specimen material needed, and differing dilution and diluent/buffer conditions. ddPCR uses amplification of transcripts, which detects very few copy numbers of the target nucleic acid. Moreover, this technology permits normalization of transcript quantity to a housekeeping gene, glyceraldehyde 3-phosphate dehydrogenase, which serves as an “internal standard” for every sample, thereby controlling for nonspecific mRNA degradation.

Additional research is needed to determine whether this panel of transcripts will predict EED in younger populations and populations different from rural African children. Analyses of fecal host transcripts should be included in clinical trials to ameliorate EED, as they may serve as a noninvasive biomarker.

## Supplementary Material

Supplemental Digital Content
