# Supplementary material for: Environmental Enteric Dysfunction Is Associated With Poor Linear Growth and Can Be Identified by Host Fecal mRNAs
Source: J Pediatr Gastroenterol Nutr. 2016 Oct 24;63(5):453–9. doi: 10.1097/MPG.0000000000001315 (PMC5084633; doi:10.1097/MPG.0000000000001315)
Supplement: Supplemental Digital Content [file jpga-63-453-s001.pdf]

SUPPLEMENTARY FIGURE 1. Comparison of the measurements of lactulose (%L) and mannitol (%M) using two methods of analysis: HPLC (high performance liquid chromatography) and LC-MSMS (liquid chromatography-tandem mass spectrometry).

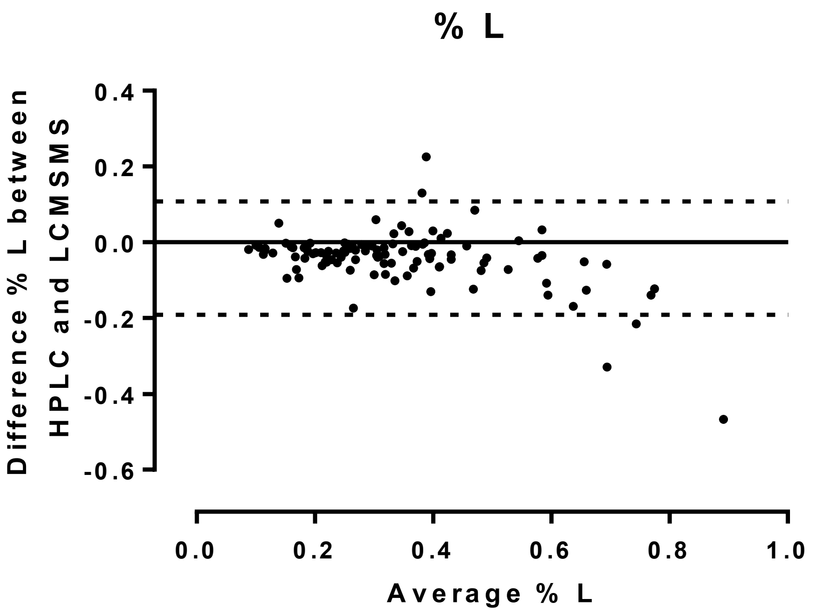

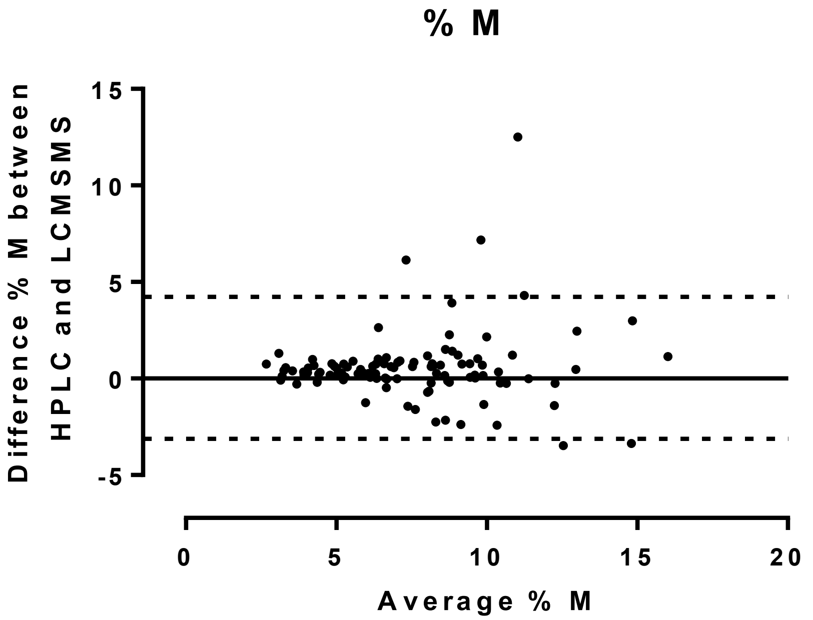

Bland-Altman plots show the differences in %L and %M, the solid horizontal line represents the mean and the dashed lines the 95% confidence interval, referred as the limits of agreement.
